# Synergistic Treatment with Ozone Water and Morpholine Fatty Acid Salts Improves Postharvest Quality in Mandarin Oranges

**DOI:** 10.3390/foods14081346

**Published:** 2025-04-14

**Authors:** Yingbin Liang, Lixin Ma, Qian Xu, Xiaoyu Tian, Li Sun, Jianrong Cai

**Affiliations:** School of Food and Biological Engineering, Jiangsu University, Zhenjiang 212013, China; a2603955191@163.com (Y.L.); lixinma99@163.com (L.M.); 13813789415@163.com (Q.X.); tianxy@ujs.edu.cn (X.T.); raulsunli@ujs.edu.cn (L.S.)

**Keywords:** mandarin orange, ozone water, morpholine fatty acid salts, synergistic treatment, postharvest quality

## Abstract

Citrus rot seriously reduces the quality of citrus, causes economic losses, and is an urgent problem for the citrus industry. Effective preservation and pretreatment methods have an important impact on the maintenance of mandarin orange quality. In this study, mandarin orange was pretreated through single and synergistic treatments with ozone water and morpholine fatty acid salts in order to assess their effects on the fruit’s physicochemical properties. First, the parameters of the ozone water treatment, including time and ozone water concentration, were optimized to determine the optimal pretreatment conditions for the subsequent mandarin orange preservation. Subsequently, the mandarin oranges subjected to different pretreatments (ozone water, morpholine fatty acid salts, ozone water + morpholine fatty acid salts, water, and blank control) were stored at 25 ± 2 °C and 75% relative humidity for 20 d to simulate retail conditions (shelf-life). Finally, the surface microbial content, firmness, weight loss, total soluble solids content, respiration rate, decay rate, and surface morphology of mandarin orange peel were assessed during the storage period. The results showed that the synergistic treatment with ozone water and morpholine fatty acid salts significantly reduced the surface microbial content (Lg CFU/g = 3.91), weight loss (2.79%), decay rate (2.5%), and firmness losses on day 20 compared to other single treatments (*p* < 0.05). Hence, synergistic treatment with ozone water and morpholine fatty acid salts is a new green mandarin orange preservation technology with promising applications in controlling postharvest diseases and extending the storage period.

## 1. Introduction

Mandarin orange (*Citrus unshiu*) is one of the main orange varieties grown in China. It has brightly colored skin, is sweet and juicy, and contains high levels of vitamins C and D. Due to microbial infestation and unsuitable storage conditions, citrus is susceptible to mildew, tissue softening, and weight loss during storage, leading to a reduction in commercial value; thus, citrus needs to be pretreated for freshness [1]. Currently, the main storage-period preservation measure for citrus is the application of chemical fungicides, such as antiseptics, and preservatives, such as imazalil, to control microbial colonization of citrus epidermis. [2,3,4]. However, the widespread application of chemical fungicides has led to the proliferation of fungicide-resistant strains, reducing the effectiveness of the treatment and generating toxic residues, posing a potential risk to the environment and human health [5,6]. An increasing awareness of food safety has prompted the search for newer, safer, and environmentally friendly alternative strategies to reduce the loss of the natural quality of the fruit and extend their storage time after harvest [7,8].

Ozone has strong oxidizing properties, can decompose itself into oxygen, and has a strong killing effect on a variety of micro-organisms such as fungi and spores [9], reducing brown rot and gray mold of fruits [10]. Ozone was granted Generally Recognized as Safe (GRAS) status by the FDA (Food and Drug Administration) in 1997 and approved for use as a sanitizer or disinfectant in food processing [11]. In addition to its sporicidal activity, ozone induces defense responses in some plants, activates fruit self-repair function, and enhances antimicrobial activity [12,13,14]. Ozone also stimulates stomatal constriction to achieve the effect of inhibiting the respiration rate of fruits and vegetables [15,16]. Ozone water treatment is also harmless to fruit and does not produce chemical residues on food surfaces [9,17].

The use of morpholine fatty acid salts is permitted in both China and the United States, the largest markets for mandarin oranges [18,19]. Morpholine acid salt is a good non-toxic antimicrobial agent for mandarin orange preservation, mainly used as a coating film for preservation [18,20]. The coating treatment effectively reduces the weight loss of fruit during storage and inhibits respiration, delaying fruit senescence to extend storage time [21]. The ozone water and morpholine fatty acid salts treatments do not interfere with each other, and there is potential for synergistic treatment. Therefore, further research is warranted to investigate whether synergistic treatment with ozone water and morpholine fatty acid salts has a better effect on the storage quality of mandarin oranges.

**Figure 1:**
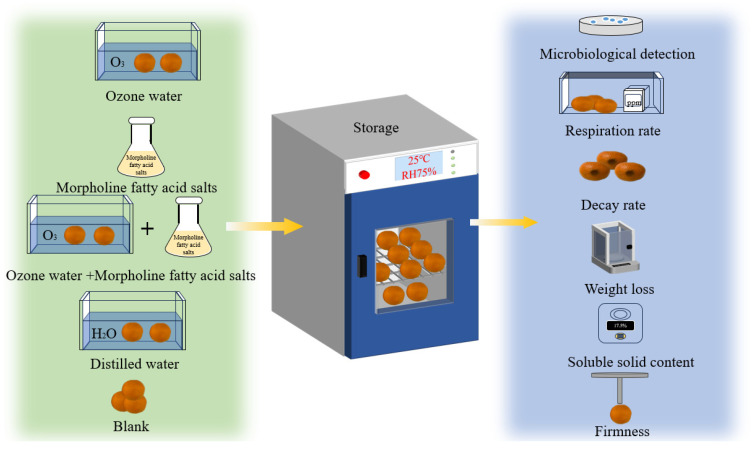
Schematic illustration of the experiment on the effect of different treatment conditions on the shelf-life quality of mandarin orange.

In this study, the synergistic treatment effects of ozone water with morpholine fatty acid salts on the development of post-harvest pathogens in mandarin oranges were analyzed, and the effects on the weight loss, decay rate, total soluble solids content, and respiration rate during storage were also evaluated, as shown in Figure 1. The purpose of this study is to evaluate the effect of collaborative treatment and to provide a new perspective in the development of an environmentally friendly and efficient method of mandarin orange preservation.

## 2. Materials and Methods

### 2.1. Ozone Water Generator Principle

To achieve the purpose of simulating natural lightning to convert oxygen into ozone, the ozone water generator adopts the corona discharge method. The principle is as follows: Sending a 220 V alternating current through the buck rectifier into a high-frequency power supply can provide a high-frequency voltage to the ozone tube so that the gas discharges, releasing a large amount of energy. The high concentration of oxygen after pretreatment produces ozone through a chemical reaction.

To increase the concentration of ozone water, the use of a micro-nano bubble-generating pump and ozone micro-nano bubble water technology will raise the ozone mass transfer efficiency and oxidation efficiency and prolong the residence time of bubbles [22,23]. The study by Gao et al. also showed that micro-nano bubbles can improve ozone utilization, increase the mass transfer rate of ozone in water, and raise the O_3_ solubility in aqueous solutions, thus improving the oxidation of O_3_ [24].

### 2.2. Ozone Water Generator Structure

**Figure 2:**
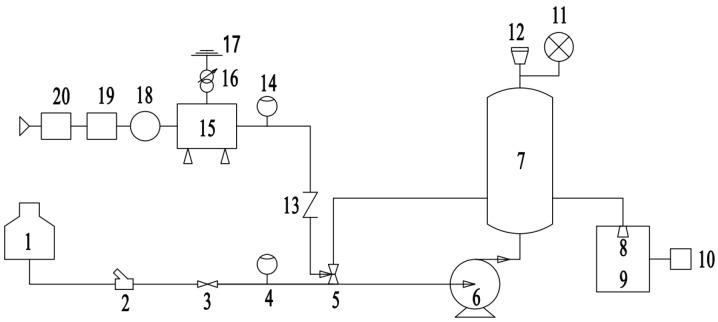
Ozone water generator process flow chart (1—container; 2—filter; 3—gate valve; 4—liquid flow meter; 5—venturi tube; 6—multi stage centrifugal pump; 7—gas–liquid stirred tank; 8—micro-nano bubble generating pump; 9—small tank; 10—TRO concentration monitor; 11—pressure gauge; 12—exhaust valve; 13—check valve; 14—gas flow meter; 15—strength ionization discharge; 16—excitation power supply; 17—ground wire; 18—cooler; 19—molecular sieve; 20—air compressor).

**Figure 3:**
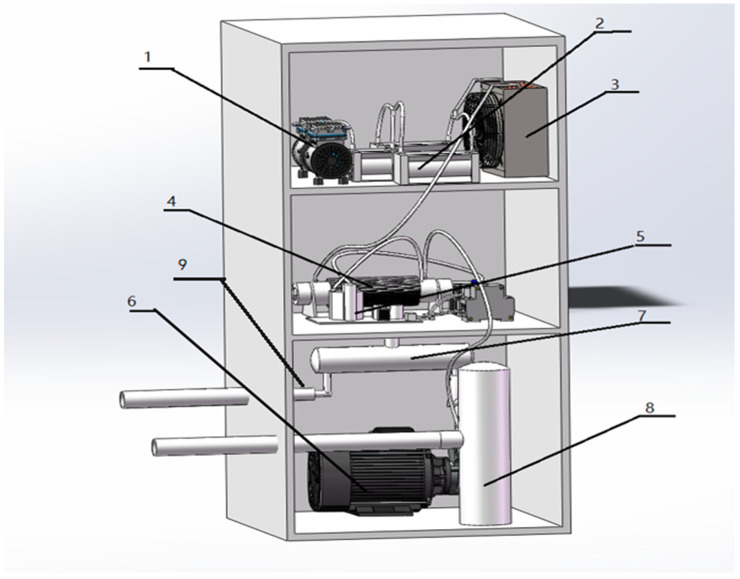
Schematic diagram of ozone water generator structure (1—air compressor; 2—molecular sieve; 3—cooler; 4—ozone generator; 5—power supply equipment; 6—gas–liquid mixing pump; 7—gas–liquid stirred tank; 8—liquid storage tank; 9—micro-nano bubble-generating pump).

High-concentration ozone water was generated by an ozone water generator. The process flow chart and structure diagram of the ozone water generator are shown in Figure 2 and Figure 3, respectively. Air was filtered through a molecular sieve to produce high-concentration oxygen, then oxygen was discharged and strongly ionized into high-concentration ozone when passing through the electrode plate. The maximum concentration of ozone can reach 100 g/m^3^, with ozone water concentration up to 20 mg/L; then, the gas–liquid mixture was realized in the mixing tank with the pressure of 0.35 MPa, the maximum flow rate of which was 5 L/min. The ozone water generator was connected to a bucket (5 L) filled with tap water. The water was continuously circulated between the ozone water generator and the bucket to produce different concentrations of O_3_ by controlling the pressure of the mixing tank. The ozone water concentration was measured by an ozone water concentration detector (CL 6587, B&C Electronics, Canat, Italy).

### 2.3. Experimental Design for Optimization of Ozone Water Treatment Conditions

The main parameters of ozone water treatment were the ozone concentration and treatment time. Three gradients of ozone water concentration of 10 mg/L, 15 mg/L, and 20 mg/L and four gradients of submergence time of 60 s, 90 s, 120 s, and 150 s were selected, and the dosage of ozone water was 23.2 L. The experiments on optimization of the conditions of ozone water treatment were conducted under an ambient environment of 25 °C, aiming to prolong the shelf-life, as exhibited in Table 1.

The treated mandarin orange samples were air-dried for three hours at room temperature. Each sample was numbered and used to track weight loss and decay, and, finally, shelf-life experiments were conducted under storage conditions of 25 ± 2 °C and RH 75% (Tianjin Teste constant temperature and humidity chamber HWS-70B) [25]. Another two repeated experiments were conducted. A total of three experiments were conducted.

During storage, fruit weight, soluble solids content (TSS), decay rate, and respiration rate were measured every 2 days. Those indicators of the samples were tracked for 10 days (2 d, 4 d, 6 d, 8 d, 10 d), and the surface microbial content of each group was examined on the day of starting storage and on the 10th day.

### 2.4. Different Pretreatment Methods and Detection

Fresh, personally picked offline mandarin oranges (*Citrus unshiu*) from local orchards in Zhenjiang, China, of decent shape (uniform and consistent); free from diseases, pests, and damage; and with consistent ripeness and color were selected as the test samples.

The experimental samples were randomly selected from the test samples. Five homogeneous groups with 40 mandarin oranges in each were set up and treated with two single treatments (ozone water or morpholine fatty acid salts) and one combined treatment (ozone water and morpholine fatty acid salts) at room temperature (20–25 °C), with distilled water immersed for 150 s treatment and no treatment as the control. The experiments were repeated twice, with a total of three experiments conducted.

The optimal parameters of the above experimental results were used as the basis for comparative experiments on mandarin oranges using the pretreatment, as shown in Table 2. The treated mandarin orange samples were air-dried for three hours at room temperature. Each sample was numbered and used to track weight loss and decay, and, finally, shelf-life experiments were conducted under storage conditions of 25 ± 2 °C and RH 75% (Tianjin Teste constant temperature and humidity chamber HWS-70B) [25].

During storage, the fruit weight, soluble solids content (TSS), decay rate, and CO_2_ content were measured every 4 days, as detailed in Section 2.5. Those indicators of the samples were tracked for 20 days (0 d, 4 d, 8 d, 12 d, 16 d, 20 d).

### 2.5. Physical and Chemical Properties of Mandarin Oranges During Storage

#### 2.5.1. Surface Microbial Content Detection Method

According to the food safety standard [GB4789.2] in China, 10 g of mandarin orange epidermis sample was weighed and placed in a sterile homogenizing cup with 90 mL of saline, and was homogenized at 8000 r/min for 1 min to make a 1:10 sample homogenate. Then, the filtered homogenate was diluted in decimal degrees to obtain a test solution that fulfills the purpose of microbiological analysis for the detection of microbial content on surfaces with different treatments. To evaluate the total viable count of the samples, the serial dilutions were plated on the standard plates of plate counting agars. Afterward, the samples were inoculated on PDA medium (Beijing Wokai Biotechnology Co., Ltd., Beijing, China) and incubated aerobically at 37 °C for 48 h, and replicates were set up. The results were expressed as Log colony-forming units per gram of peel (Log_10_ CFU/g).

#### 2.5.2. Weight Loss Rate Detection Method

The weight loss of each group of samples was monitored using an electronic balance (Ji Ming Weighing and Calibration Equipment Co., Ltd., Hangzhou, China, accuracy 0.01 g). For each group, 40 fruits were numbered, and the same fruits were weighed at each time interval to calculate the percentage difference in weight. This was repeated three times to calculate the average value.

The mass loss was given by the equation [26]:

*WL_t_* (%) = (*W*_0_ − *W_t_*)/*W*_0_ × 100 (1)

where *WL_t_* was the percentage weight loss at moment *t*; *W*_0_ was the initial sample weight; and *W_t_* was the sample weight at moment *t*.

#### 2.5.3. Soluble Solids Content Detection Method

The soluble solids content of each group of the three random mandarin orange samples was measured using a handheld refractometer (LH-T20, Shanghai Basis Instruments Co., Ltd., Shanghai, China) and repeated three times along with the calculation of the average value.

#### 2.5.4. Respiration Rate Detection Method

Each group of samples was placed in a sealed box (49.5 cm × 38.5 cm × 24.5 cm) for 30 min, and the concentration of CO_2_ in the box was detected by a homemade fruit and vegetable storage environment monitor; after the detection, the residual gas in the sealed box was cleaned by forced air exchange, and the experiment was repeated twice to record the measured values and calculate the average increase in CO_2_ gas.

The respiration rate was determined using the following formula:

*V*_*CO*_2__ (mg kg^−1^ s^−1^) = (*m_t_* − *m*_0_)/*t* (2)

where *V*_*CO*_2__ was the average rate of carbon dioxide production at time *t*; *m*_0_ was the initial carbon dioxide weight; and *m_t_* was the carbon dioxide weight at time *t*.

#### 2.5.5. Decay Rate Detection Method

The rate of decay was determined by counting mold-infected and slightly decayed fruit of each group of 40 random mandarin oranges, and the results are expressed as a percentage of decayed fruit [12,26].

#### 2.5.6. Firmness Detection Method

Firmness was determined by using a texture analyzer (HADPNI GY-4, China). The fruit firmness of three random mandarin orange samples from each group was recorded using an 8 mm flat probe (area of 50.27 mm^2^) and by measuring the maximum penetration of the peel with a probe speed of 12 mm per second. Two measurements were obtained on two opposite equatorial fruit zones. The results were reported as the peak force in Newtons (N).

#### 2.5.7. Morphological Properties Detection Method

The morphological mandarin orange peel samples were obtained on a scanning electron microscope (S-3400N Hitachi Tokyo, capital of Japan) at 15.0 kV. Approximately 2 mg of the samples was sprinkled on a conductive adhesive and coated with gold for 30 s (25 mA, 2 × 10^−4^ MPa).

### 2.6. Statistical Analysis

The results were statistically analyzed using statistical software (IBM SPSS Statistics 25 for Windows), the data were analyzed using the one-way analysis of variance (ANOVA), and the significant differences were detected by the Duncan’s test (*p* < 0.05).

## 3. Results and Discussion

### 3.1. Optimization of Conditions for Ozone Water Treatment

**Figure 4:**
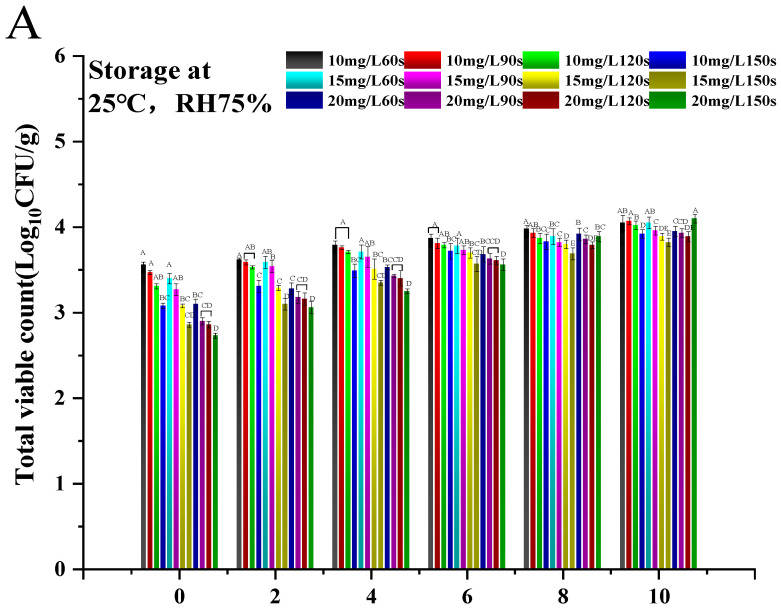

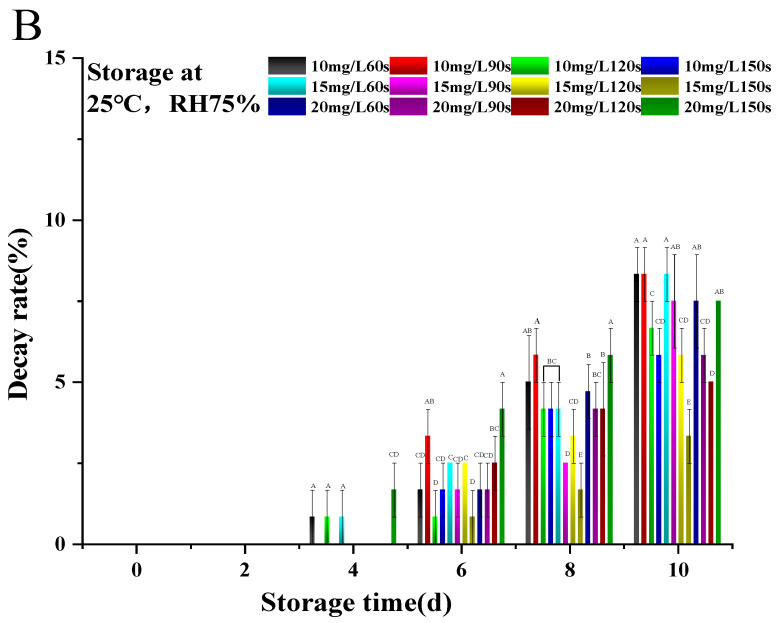
Total number of colonies (**A**) and decay rate (**B**) of mandarin oranges after treatment with different ozone water concentrations and time during shelf-life for 10 d at 25 °C 75%. Data are mean ± standard deviation (SD) of three replicates. Different letters are significantly different using ANOVA at *p* < 0.05.

The results of the optimization of ozone water treatment conditions are shown in Figure 4 and Figure S1. Data were not significantly (*p* > 0.05) different between treatment groups, including weight loss (Figure S1A), soluble solids content (Figure S1B), respiration rate (Figure S1C), and firmness (Figure S1D). In terms of weight loss, 10 mg/L for 60 s, 15 mg/L for 60 s, and 15 mg/L for 150 s were significantly lower than the other groups at 10 d (*p* < 0.05), but there was no significant difference overall (*p* > 0.05). This may be because the concentration gradient and treatment time gradient did not differ much due to the pursuit of the optimal ozone water treatment conditions in this experiment, which resulted in no significant difference found in these four experimental parameters. However, the 15 mg/L 150 s treatment group had a significant advantage (*p* < 0.05) in reducing the decay rate (Figure 4B) and surface microbial content (Figure 4A). The decay rate of the 15 mg/L 150 s treatment group was 3.33% at 10 d, which was much lower than the average decay rate of 6.66% in the present experiments; therefore, the 15 mg/L 150 s treatment group was considered to be the most effective in the screening experiment of treatment conditions. Therefore, in the screening experiment of treatment conditions, the 15 mg/L 150 s treatment group was considered to have the best effect, the concentration of ozone water treatment was selected as 15 mg/L, and the treatment time was selected as 150 s in the subsequent experiments.

### 3.2. Analysis of Physicochemical Properties and Shelf-Life of Mandarin Oranges After Different Treatments

#### 3.2.1. Surface Microbial Content

**Figure 5:**
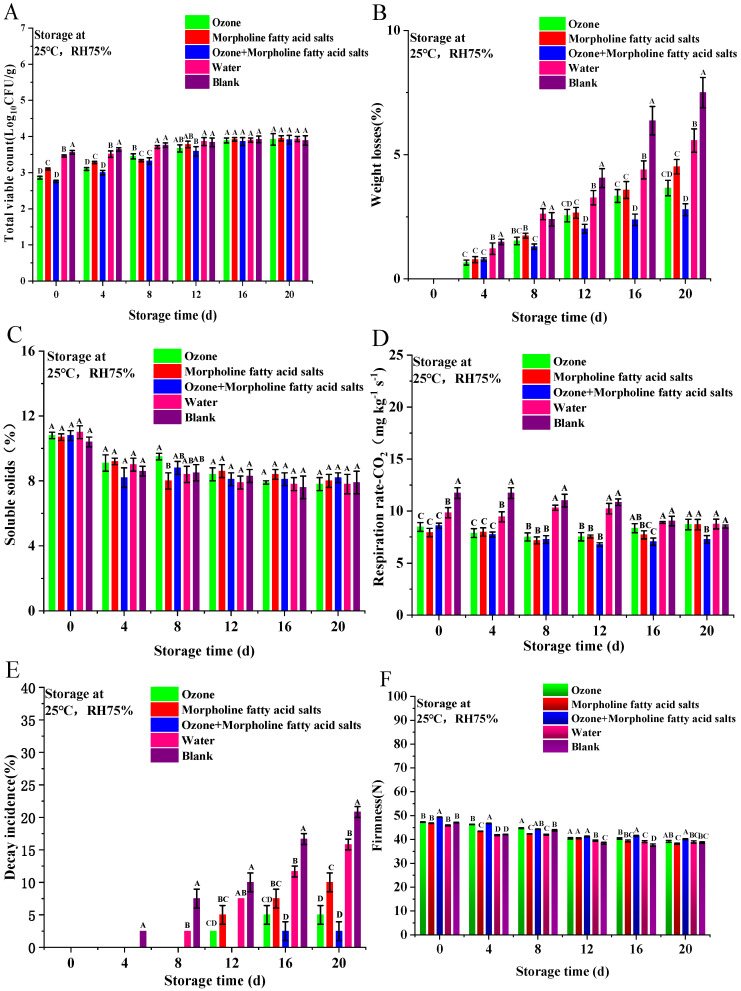
Total number of colonies (**A**), weight loss (**B**), soluble solids content (**C**), respiration rate (**D**), decay rate (**E**), and firmness (**F**) of mandarin oranges after different pretreatments during shelf-life for 20 d at 25 °C RH75%. Data are mean ± SD of three replicates. Different letters are significantly different using ANOVA at *p* < 0.05.

The statistics of the surface microbial content of mandarin oranges are shown in Figure 5A. Storage time had a significant effect on the surface microbial content of mandarin oranges, and the longer the storage time, the higher the surface microbial content, which was caused by the gradual proliferation of surface micro-organisms (*p* < 0.05). Previous experiments to screen the optimal ozone water treatment conditions have been able to determine the optimal parameter for ozone water treatment in 10 d based on indicators such as spoilage rate. Subsequent experiments used this optimal parameter for the effect of different preservation pretreatments on the postharvest quality of citrus. Data were collected for 20 days for this procedure. Ozone water treatment was significantly better than all other groups at 0 to 4 d, indicating that ozone was more effective in killing and controlling micro-organisms at 0 to 4 d. At 4 d to 8 d, the morpholine fatty acid salts treatment was significantly better than the other groups, which indicated that the bacterial inhibitory effect of its coating was more durable. At 16 d, the surface microbial content of the ozone water + morpholine fatty acid salts treated group (Lg _CFU/g_ = 3.86) was significantly lower than that of the other groups, indicating that the composite treatment had better bacterial inhibition and synergistic treatment advantages. The results are consistent with the results of the previous studies [27,28,29]. According to their studies, ozone water can kill the spores on the fruit surface, and the morpholine fatty acid salts contain antibacterial components that will also inhibit microbial reproduction. Additionally, according to the analysis of experimental data, the antibacterial effect of ozone water is slightly better than morpholine fatty acid salts, and the washing effect of distilled water may also have a certain effect of removing micro-organisms from the mandarin orange surface [30,31]. The findings from this experiment are also in agreement with the results of Eva Fathul Karamah regarding the storage of tofu in a refrigerator at 8 °C for seven days after ozone water treatment. Their study concluded that ozone water treatment could be effective in killing micro-organisms, with the longer the ozone water treatment time, the better the sterilization effect. In the present experiment, the difference in microbial content between ozone water and morpholine fatty acid salts treatments and the other groups narrowed down after 4 d. This may be due to the gradual weakening of the ozone water and morpholine fatty acid salts’ antibacterial effect with the extension of storage time.

#### 3.2.2. Weight Loss

From an economic point of view, weight loss seriously affects mandarin oranges’ economic value, as they are currently sold by weight. The transpiration of water and loss of sugar will also reduce the appearance and internal quality of mandarin oranges, which decreases their attraction for consumers.

The weight loss of mandarin orange samples from different treatment groups is shown in Figure 5B. Storage time had a significant effect on the weight loss rate of mandarin oranges; the longer the storage time, the higher the weight loss rate (*p* < 0.05). In the early stage, the weight loss rate of the experimental groups at 4 d was lower than that in the control group (ozone water: 0.66%; morpholine fatty acid salts: 0.78%; ozone water + morpholine fatty acid salts: 0.78%). Both ozone water and morpholine fatty acid salts treatments were beneficial in reducing fruit weight loss during storage in mandarin oranges, which was consistent with the results of other studies [6,32,33]. However, there were no differences in the data between the single and synergistic treatments, probably because the single treatment was effective at the beginning of storage and the treatment effect did not diminish. There was no difference in weight loss between the ozone water and morpholine fatty acid salts treatments at 4 d, 8 d, 12 d, and 16 d, demonstrating that these two treatments were similar in reducing the weight loss rate in mandarin oranges at the beginning of storage. At 20 d, the weight loss results of ozone water treatment were borderline with that of morpholine fatty acid salts treatment (ozone water: 3.66%; morpholine fatty acid salts: 4.52%), which proved that ozone water treatment was slightly better than morpholine fatty acid salts treatment in reducing weight loss under this experimental condition for the loss of wax coating layer during storage, resulting in the diminishing effect of the morpholine fatty acid salts treatment. However, the weight loss of the ozone water + morpholine fatty acid salts synergistic treatment (2.79%) was lower than those of other groups, which proved the superiority of the synergistic treatment.

Mandarin orange weight loss is mainly due to fruit respiration and transpiration. The experimental results showed that both ozone water and morpholine fatty acid salts treatments reduced weight loss in mandarin oranges, and the synergistic treatment of ozone water + morpholine fatty acid salts further reduced weight loss in mandarin oranges during storage. Swarajya Laxmi Nayak et al. used 0.1 ppm ozone water to treat strawberries and obtained similar findings [34]. The results of his study showed that ozone water treatment inhibited the respiration of mandarin oranges and delayed the natural dewaxing process of the fruit. Additionally, the content of several metabolites increased one day after ozone water treatment, especially soluble sugars and organic acids. These metabolites may play a regulatory role in ozone-induced fruit resistance, helping to reduce fruit weight loss.

#### 3.2.3. Soluble Solids Content

Soluble solids content of mandarin orange samples from different treatment groups is shown in Figure 5C. Storage time had a significant effect on the soluble solids content of mandarin oranges, and the longer the storage time, the lower the soluble solids content due to the loss of fruit nutrients after picking (*p* < 0.05). The soluble solids content of mandarin orange samples decreased during storage, but no differences were observed between groups (*p* > 0.05), which is in agreement with the results of Garcia-Martin et al. [6]. They used treatments ranging from 1.6 to 60 mg/kg of ozone at 5 °C for 28 days and stored at 20 °C, and no differences were found in soluble solids content. The reason might relate to tissue glycation and higher water loss in the control groups during storage, which did not result in differences in soluble solids content between treatments.

#### 3.2.4. Respiration Rate

The respiration rate of mandarin orange samples from different treatment groups was shown in Figure 5D. The storage time had a significant effect on the respiration rate of mandarin oranges, which was higher at the beginning of storage and then gradually decreased with the increase in storage time due to nutrient loss; mandarin oranges gradually enter a state of low physiological response after picking (*p* < 0.05). However, the highest respiration rate was observed on the 20th day of the storage period, which was caused by the aggravation of mandarin orange fruit decay and the rapid propagation of micro-organisms. There was a difference between the treatment and control groups at 0 d, 4 d, 8 d, and 12 d, with the samples in the treatment group better maintaining their quality in the early stages of storage, and there was little loss of treatment effect. There was a difference between the blank control group and the distilled water control group at 0 d and 4 d, which was related to the fact that the washing with distilled water still had a cleaning effect, reducing the natural loss of the waxy layer of the fruit and avoiding elevated respiration rates. The rate of respiration of the other treatment groups was higher than the ozone water + morpholine fatty acid salts treatment groups at 16 and 20 d. The ozone water + morpholine fatty acid salts treatment reduced the respiration rate of the mandarin orange samples (20 d: 7.2672 mg kg^−1^ s^−1^). Liu, Z et al. used ozone water fumigation of sweet cherries at low temperatures and observed by scanning electron microscopy that ozone water stimulated stomatal constriction and reduced the respiration rate [35]. A study by Yang, Luzhu et al. showed that fruit wax could form a thin film on the fruit surface that prevents water loss and shrinkage, thus reducing transpiration and respiration intensity [36]. The studies of Khalifa et al. and Tesfay et al. also found the same results [37,38]. The experimental results showed these treatments (ozone water; morpholine fatty acid salts; ozone water + morpholine fatty acid salts) resulted in respiratory inhibition at 0, 4, 8, and 12 d, which was consistent with the findings of previous studies [25]. Due to the elevated effect of the combined treatment, the ozone water + morpholine fatty acid salts treatment was the most effective in this experiment.

It is worth noting that the effects of ozone water and morpholine fatty acid salts treatments on mandarin oranges gradually weakened with storage time, and the respiration rate was no longer different from that of the control group at 20 d, which might be caused by the gradual failure of ozone water treatment [39].

#### 3.2.5. Decay Rate

Post-harvest rot is the most serious problem facing the mandarin orange industry [25]. In terms of the incidence of decay, the effect of different treatments on mandarin oranges was shown in Figure 5E. Storage time has a significant effect on the decay rate of mandarin oranges; the longer the storage time, the higher the decay rate (*p* < 0.05). Only the blank control group showed decay at 4 d, which may be related to the fact that this group was not treated and had more microbial pathogens attached to the surface than the washed distilled water control group, leading to early decay of the mandarin orange samples. Only the ozone water + morpholine fatty acid salts synergistic treatment group did not experience decay at 8 d and 12 d. The groups treated with ozone water and ozone water + morpholine fatty acid salts had the two lowest decay rates during the experiment, which was attributed to the bactericidal effect of ozone water and its ability to increase the antioxidant capacity, activate the self-repair mechanism, and delay the fruit decay of mandarin oranges [12]. In terms of decay rate reduction, morpholine fatty acid salts treatment was less effective than ozone water treatment but still better than the control groups, because there is the presence of certain antibacterial components in morpholine fatty acid salts as well [14]. At 12 d, 16 d, and 20 d, synergistic treatment and single treatment reduced the decay rate (ozone water: 2.5%, 5%, 5%; morpholine fatty acid salts: 5%, 7.5%, 10%; ozone water + morpholine fatty acid salts: 0%,2.5%,2.5%).

**Figure 6:**
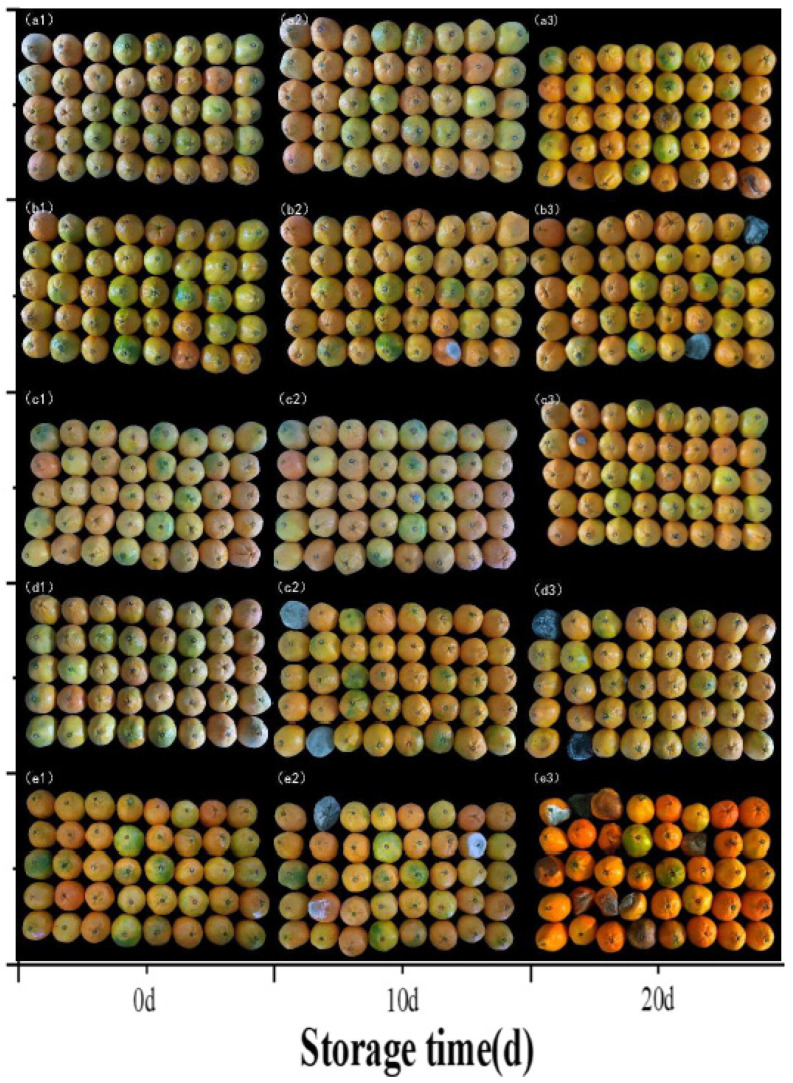
Physical images of mandarin orange during the storage period (0 d, 10 d, 20 d) with different pretreatments: (**a**) ozone water; (**b**) morpholine fatty acid salts; (**c**) ozone water + morpholine fatty acid salts; (**d**) distilled water; (**e**) blank.

Images of the mandarin orange samples with different treatments during storage are shown in Figure 6. Considerable decay of the mandarin orange samples can be seen in the blank control group, with the decay rate higher than in the distilled water control group because pf the washing effect of water on the fruit’s surface, which could delay the decay to some extent. Ozone water treatment and ozone water + morpholine fatty acid salts synergistic treatment were the most effective in delaying decay, with the samples remaining in a slightly better condition than those treated with only morpholine fatty acid salts. Qixian Wu treated satsuma mandarin orange with 2 μg/L ozone gas for 5 h. The decay rate was reduced by 15.5% compared with the control group [25].

The results showed that the synergistic treatment had an effect on delaying and reducing the degree of decay of mandarin orange samples during storage. On the one hand, it may be that ozone water treatment inhibits mold infestation and reduces spoilage rates [14]. On the other hand, it may be noted that morpholine fatty acid salts contain antimicrobial components that are also effective in inhibiting microbial growth [18].

#### 3.2.6. Firmness

In terms of the firmness, the effect of different treatments on mandarin oranges is shown in Figure 5F. Storage time has a significant effect on the hardness of mandarin oranges; the longer the storage time, the lower the hardness due to the gradual softening of the mandarin orange tissues after picking. Ozone had a significant effect on the firmness of mandarin orange fruits for the first eight days of storage. The mandarin orange samples from the ozone water treatment group and the ozone water + morpholine fatty acid salts treatment group lost their hardness slower than the mandarin orange samples from the distilled water control group and the blank control group, which proved that the ozone water treatment could indeed delay the loss of hardness of the fruits, that there was no significant difference between the morpholine fatty acid salts treatment group and the control group, and that the morpholine fatty acid salts treatment might not have a significant effect on the hardness of the mandarin orange samples. However, the effect of ozone water treatment was gradually lost with the increase in storage time, and the difference between the hardness of mandarin orange samples in the ozone water and ozone water + morpholine fatty acid salts treatment groups and the other groups gradually narrowed, as evidenced by the results of a related study by Juan Francisco García-Martín et al. [6].

#### 3.2.7. Morphological Properties

**Figure 7:**
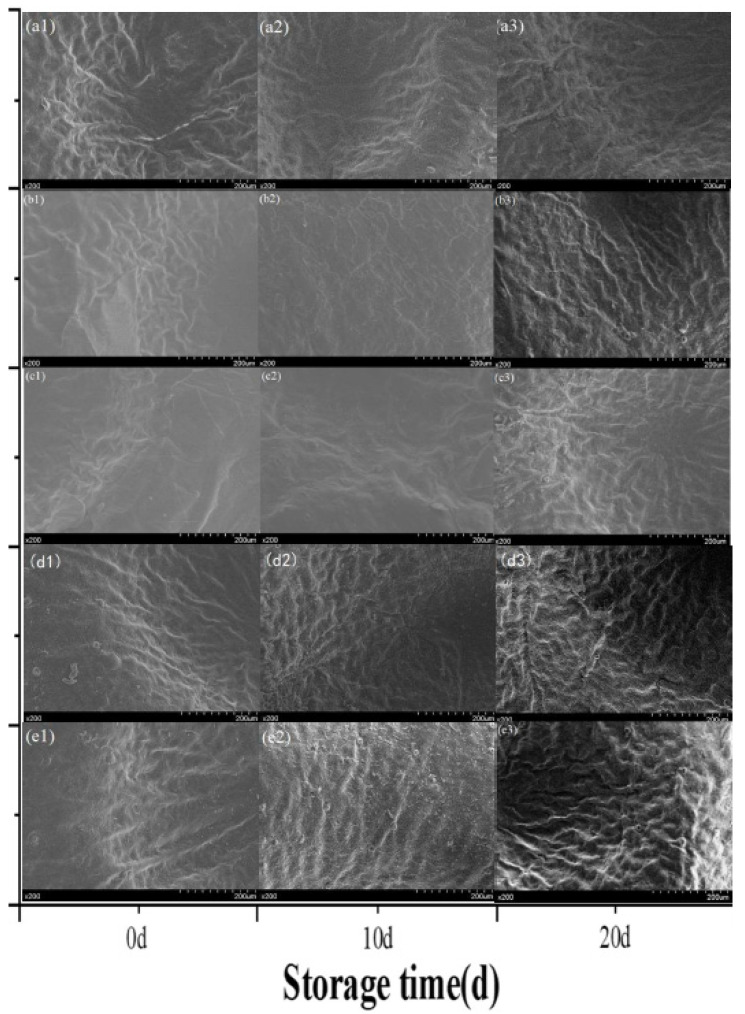
SEM images of mandarin orange peels during the storage period (0 d, 10 d, 20 d) with different pretreatments: (**a**) ozone water; (**b**) morpholine fatty acid salts; (**c**) ozone water + morpholine fatty acid salts; (**d**) distilled water; (**e**) blank. For example, (**a1**) is an image of the ozone water treatment group 0 d.

SEM images of mandarin orange peel SDF samples at ×200 magnification are shown in Figure 7. At 0 d, no significant differences were found in the epidermis of the samples from the ozone water treatment group, distilled water control group, and blank control group, and the epidermises of the morpholine fatty acid salts treatment group and ozone water + morpholine fatty acid salts treatment group were in close proximity to each other, with the applied morpholine fatty acid salts forming a protective film on the mandarin orange epidermis. At 10 d, the epidermal conditions of the mandarin orange samples from the ozone water group, morpholine fatty acid salts treatment group, and ozone water + morpholine fatty acid salts treatment group were significantly better than that of the control group, and it is possible that ozone stimulates the resilience mechanisms of mandarin orange, delaying fruit senescence and enhancing enzyme activity, and that the protective layer of morpholine fatty acid salts is effective in slowing epidermal water loss [14]. The epidermis of the control group mandarin orange samples was wrinkled to a certain extent due to the loss of water, among other reasons. At 20 d, the epidermal film of the samples from the morpholine fatty acid salts treatment group and the ozone water + morpholine fatty acid salts treatment group was shed to some extent, and the ozone treatment effect of the ozone water treatment group was gradually lost; however, the epidermal condition was still better than that of the control group, which suffered considerable water loss, with the epidermal condition of the ozone water + morpholine fatty acid salts treatment group being the best.

## 4. Conclusions

In this study, we investigated the effects of ozone water and morpholine fatty acid salts synergistic treatment on the microbial control and postharvest quality of mandarin oranges. Ozone water and morpholine fatty acid salts can inhibit mandarin orange decay and weight loss during storage and can effectively inhibit respiration; ozone water treatment’s effect is better, but the morpholine fatty acid salts treatment effect lasts longer. When ozone water is synergistically treated with morpholine fatty acid salts, the treatment’s effect is elevated and has the advantages of both. The results show that the synergistic treatment of ozone water with morpholine fatty acid salts is superior to any single treatment in terms of the weight loss, surface microbial content, decay rate, respiration rate, and epidermal condition of mandarin oranges at the early stage of storage, demonstrating the feasibility of this treatment for mandarin orange preservation.

## Figures and Tables

**Table 1:** Ozone water screening experiment treatment conditions.

No.	Pretreatment Method	Description
1	Ozone water 1	Continuous immersion of 10 mg/L ozone water for 60 s
2	Ozone water 2	Continuous immersion of 10 mg/L ozone water for 90 s
3	Ozone water 3	Continuous immersion of 10 mg/L ozone water for 120 s
4	Ozone water 4	Continuous immersion of 10 mg/L ozone water for 150 s
5	Ozone water 5	Continuous immersion of 15 mg/L ozone water for 60 s
6	Ozone water 6	Continuous immersion of 15 mg/L ozone water for 90 s
7	Ozone water 7	Continuous immersion of 15 mg/L ozone water for 120 s
8	Ozone water 8	Continuous immersion of 15 mg/L ozone water for 150 s
9	Ozone water 9	Continuous immersion of 20 mg/L ozone water for 60 s
10	Ozone water 10	Continuous immersion of 20 mg/L ozone water for 90 s
11	Ozone water 11	Continuous immersion of 20 mg/L ozone water for 120 s
12	Ozone water 12	Continuous immersion of 20 mg/L ozone water for 150 s

**Table 2:** Different treatment conditions.

No.	Pretreatment Method	Description
1	Ozone water	Continuous immersion of 15 mg/L ozone water (23.2 L) for 150 s
2	Morpholine fatty acid salts	Apply morpholine fatty acid salts (20 g/kg Jianling Laboratory) thinly and evenly on the surface of the mandarin oranges
3	Ozone water + morpholine fatty acid salts	Continuous shower with 15 mg/L of ozone water for 150 s and then apply morpholine fatty acid salts to the surface of mandarin oranges in a thin and even layer
4	Distilled water	Continuous immersion in distilled water for 150 s
5	Blank	No treatment

## Data Availability

The original contributions presented in the study are included in the article, further inquiries can be directed to the corresponding author.

## Supplementary Materials

The following supporting information can be downloaded at: https://www.mdpi.com/article/10.3390/foods14081346/s1. Figure S1: Weight loss (A), soluble solids content (B), respiration rate (C), and firmness (D) of mandarin oranges after treatment with different ozone water concentrations and time during shelf-life for 10 d at 25 °C 75%.
