# Supplementary material for: Synergistic Treatment with Ozone Water and Morpholine Fatty Acid Salts Improves Postharvest Quality in Mandarin Oranges
Source: Foods. 2025 Apr 14;14(8):1346. doi: 10.3390/foods14081346 (PMC12027277; doi:10.3390/foods14081346)
Supplement: Supplementary file 1 [file foods-14-01346-s001.zip › foods-3562027-supplementary.pdf]

Supplementary Material

Synergistic treatment with ozone water and morpholine fatty acid salts improves postharvest quality in mandarin oranges

Yingbin Liang[a], Lixin Ma[a], Qian Xu[a], Xiaoyu Tian[a], Li Sun[a], Jianrong Cai[a*]

[a] *School of Food and Biological Engineering, Jiangsu University, Zhenjiang 212013, China*

*Corresponding author at School of Food and Biological Engineering, Jiangsu University, 212013 Zhenjiang, China

Email address: jrcai@ujs.edu.cn (J.R. Cai)

Results and discussion

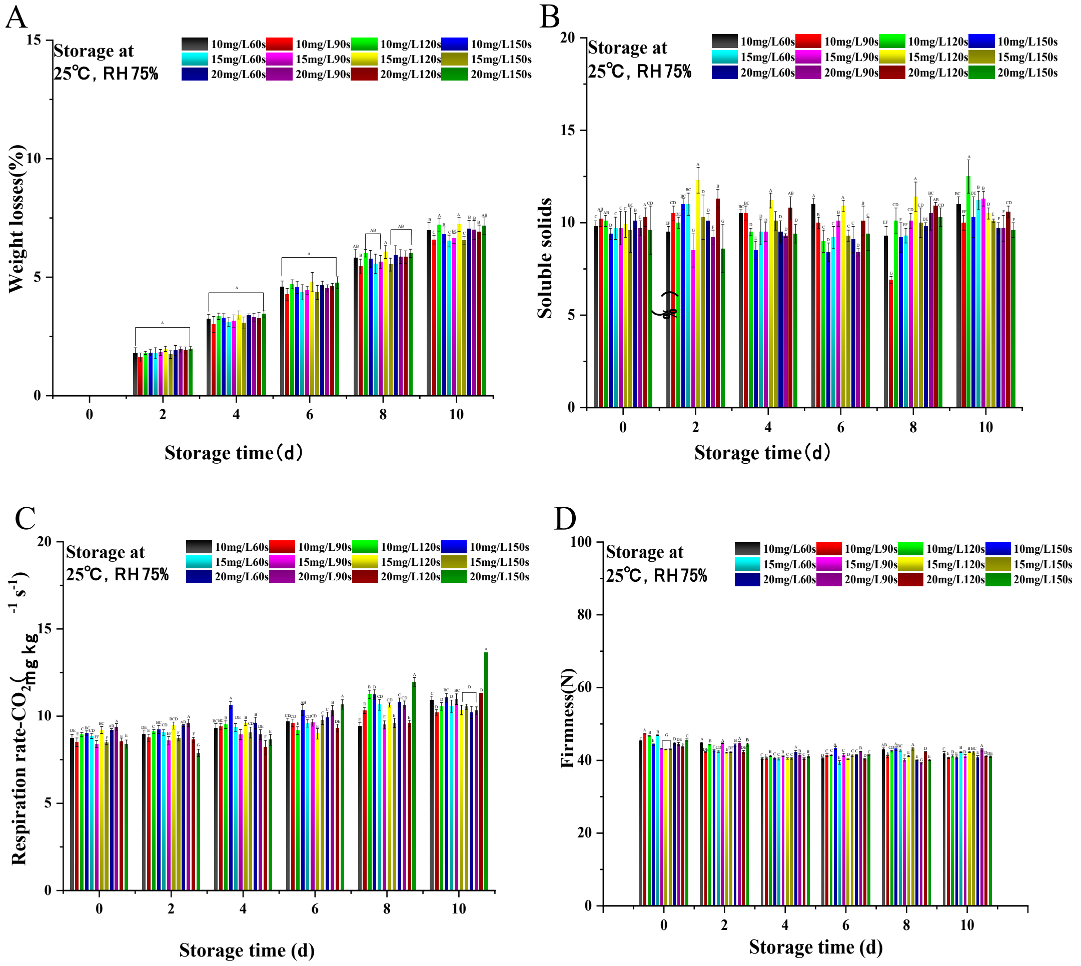

Figure. S1. Weight loss (A), soluble solids content (B), respiration rate (C), and firmness (D) of mandarin oranges after treatment with different ozone water concentrations and time during shelf-life for 10 d at 25 °C 75 %. Data are mean ± standard deviation (SD) of three replicates. Different letters are significantly different using ANOVA at $p < 0.05$.
